# Update on Viral Gene Therapy Clinical Trials for Retinal Diseases

**DOI:** 10.1089/hum.2022.159

**Published:** 2022-09-16

**Authors:** Shun-Yun Cheng, Claudio Punzo

**Affiliations:** ^1^ Departments of Ophthalmology and Visual Sciences, University of Massachusetts Medical School, Worcester, Massachusetts, USA.; ^2^ Departments of Gene Therapy Center, University of Massachusetts Medical School, Worcester, Massachusetts, USA.; ^3^ Departments ofNeurobiology, University of Massachusetts Medical School, Worcester, Massachusetts, USA.

**Keywords:** clinical trials, retinal gene therapy, retinal degeneration

## Abstract

In 2001, the first large animal was successfully treated with a gene therapy that restored its vision. Lancelot, the Briard dog that was treated, suffered from a human childhood blindness called Leber's congenital amaurosis type 2. Sixteen years later, the gene therapy was approved by the U.S. Food and Drug Administration. The success of this gene therapy in dogs led to a fast expansion of the ocular gene therapy field. By now every class of inherited retinal dystrophy has been treated in at least one animal model and many clinical trials have been initiated in humans. In this study, we review the status of viral gene therapies for the retina, with a focus on ongoing human clinical trials. It is likely that in the next decade we will see several new viral gene therapies approved.

## INTRODUCTION

Gene therapy has been explored for decades with the hope to treat a wide range of genetic diseases.^1^ It involves the delivery of genetic material as an intervention to modify the transcriptome of living cells. The safety and efficacy of a gene therapy are particularly important to assess when the treatment is designed to be long-lasting.^1^ One of the factors that determine the efficiency of a gene therapy is how well target cells can be transduced. This step involves the transport/delivery of the genetic material to the target cells, followed by the uptake and the successful use of the genetic material by the target cells. A delivery system that carries the genetic material can be divided into two broad groups as follows: viral based and nonviral based.^2^

Each delivery system has its advantages and limitations depending on the disease that needs to be treated. In this study, we focus on viral delivery systems for the treatment of retinal diseases.

Eyes are an ideal target for gene therapy treatment. They are easily accessible and the transparent nature of the anterior part of the eye allows for the use of different noninvasive examination techniques to assess the integrity and function of the retina, which is the primary target tissue for many eye gene therapies. This allows not only to diagnose and identify retinal diseases but also to evaluate the efficacy and toxicity of a treatment. In addition, visual function can also be directly report by patients, can be evaluated on reading charts, or can be assessed by behavioral tests such as walking through a maze.

The retina is a well-studied tissue and there are >200 genes associated with retinal diseases that when mutated cause vision loss and retinal degeneration.^3^ Importantly, the retina is immune privileged due to the presence of two retinal blood barriers in the eye. Thus, viral gene therapy is more feasible since the immune response is suppressed.^4^

The administration route also plays an important role in the success of a therapy and in eliciting an immune response. The two common injection routes for viral delivery to the retina are intravitreal or subretinal injection. Intravitreal injection delivers the virus close to the inner layers of retina. This route is preferred when targeting ganglion cells and inner nuclear layer (INL) cells. Subretinal injections are preferred when treating mutations in photoreceptor (PR) or retinal-pigmented epithelium (RPE) expressed genes. Subretinal injections can also target INL cells. The advantage of subretinal injections is that the injection site is behind the retinal–blood barriers.

The disadvantage is, it requires a retinal detachment and the formation of a subretinal bleb at the injection site, which may further affect retinal integrity in patients who already have preexisting retinal degeneration due to the disease condition they suffer from. The procedure requires a retinal surgeon, and is more invasive and riskier. Intravitreal injections are noninvasive and can be performed by most ophthalmologists, but are more likely to elicit a mild immune response. Intravitreal injections are not only used to target ganglion cells but are also often used to target any inner retinal cells to produce secreted proteins if PRs or RPE cells do not need to be targeted. The reason for this is the safety profile of the intravitreal injection technique itself.

In recent years, new serotypes have been developed that can transduce PRs when delivered by an intravitreal injection.^5,6^ The goal of identifying such serotypes is to circumvent the more difficult and riskier subretinal injection procedure and to transduce PRs over an area larger than can be created with a subretinal bleb. This goal is also helped by a newly developed less invasive procedure, suprachoroidal injection, which has been shown to also transduce PRs.^7^ The procedure can be performed with catheters, needles, and microneedles.^7^ The suprachoroidal space provides a greater coverage of gene transfer across the eye.^8^ However, an immune response, as well as fast clearance of the virus, may occur due to the lack of a blood barrier and the porous nature of the choroidal vasculatures.^7^

The reason for suprachoroidal injections not being used more frequently yet in clinical trials is the novelty of the procedure. Successful trial results will undoubtedly shift the field in the future from subretinal injections to suprachoroidal injections.

In 2016, we reviewed in “Advances in Gene Therapy for Diseases of the Eye” therapeutic strategies for different PR-related diseases.^9^ In 2017, the U.S. Food and Drug Administration (FDA) approved the first *in vivo* gene therapy product (Luxturna) to treat an inherited retinal dystrophy (IRD) caused by biallelic mutations in the *Retinal-pigmented epithelium 65* (*RPE65*) gene, which lead to visual impairment in both children and adults. Since then, many gene therapy clinical trials have emerged for the different types of IRDs such as Leber congenital amaurosis (LCA), Leber hereditary optic neuropathy (LHON), Stargardt's disease, and retinitis pigmentosa (RP). Some clinical trials have also started to use mutation-independent targets to treat other retinal diseases such as neovascular age-related macular degeneration (nAMD) and diabetic retinopathy. In this study, we review ongoing viral gene therapy strategies for the retina, including delivery vectors and approaches, with a focus on the existing clinical trials.

## INHERITED RETINAL DYSTROPHIES

The cell types most affected by mutations in genes that cause IRDs are PRs, RPE cells, and ganglion cells. Mutations that affect PRs progress as a rod-cone dystrophy (RCD), cone-rod dystrophy (CRD), or cone dystrophy (CD), if the mutation is in a cone-specific gene. The nomenclature denotes which of the two PR cell types starts dying first. Because in human and mouse, rod loss always leads to cone loss,^10–14^ RCDs can also be caused by mutations in rod-specific genes only, which contrasts CRD where the mutation is in a gene that is expressed in both PR cell types. Mutations that affect RPE function and/or survival will always lead to PR death (RCD or CRD) because the RPE supports PR function and metabolism.^15^

Retinal disease nomenclature can be confusing as different mutations within the same gene can cause different diseases (pathologies), while one disease can be caused by many different genes. For example, RP is associated with more than 40 genes that affect PR and/or RPE function.^16^ The disease name describes a fundus pathology that was interpreted to be an inflammation of the retina (itis: inflammation) with pigment deposition. The disease stages attributed to RP are consistent with an RCD where individuals develop first night blindness (loss of rod PRs), followed by loss of peripheral vision (tunnel vision: loss of peripheral cone PRs) and then complete blindness (loss of macular cones).^17^ Although this is consistent with a mutation in a rod-specific gene, the late-stage fundus pathology of a CRD will resemble that of an RCD.

In contrast, mutations in the gene *CEP290*, which a cilia protein expressed in PRs and other ciliated cells, can cause a wide range of diseases, including nonsyndromic LCA10 as well as Bardet–Biedl syndrome,^18^ Joubert syndrome,^19^ Senior–Loken syndrome,^20^ and Meckel–Gruber syndrome.^21^

In this study, we discuss only clinical gene therapy trials that use viral vectors for gene transfer. Viral vectors utilize the natural infectious characteristics of viruses to enter cells and deliver the desired genetic material to the nucleus.^2^ The viral genome is replaced with the desired transgene cassette to transduce cells and express the correct disease-related gene product. An ideal delivery system should be able to contain the size of the correcting gene, deliver the gene into the desired cell types, and provide expression of the gene product at the therapeutic level.^22^ We discuss the two vectors, lentivirus and recombinant adeno-associated-virus (rAAV), that have so far been used in human clinical trials for the treatment of retinal disorders.

In general, lentiviruses are the virus of choice for large genes due to the limited packing capacity of rAAV vectors of ∼4.7 kb, while rAVV vectors are preferred for small genes due to their safety profile. For simplicity we created three major subgroups for each vector: PR and RPE diseases and related genes, INL and ganglion cell layer (GCL) diseases, and mutation-independent disease approaches. We highlight the disease name and gene involved within each subgroup.

## LENTIVIRUS

The lentivirus is a retrovirus that transduces its RNA genome into host cells upon infection and reverse transcription.^23^ Unlike other retroviruses, lentiviruses can transduce postmitotic cells, making it the retroviral vector of choice for gene therapy of postmitotic PRs.^24^ Furthermore, lentiviruses have been successfully used to deliver large transgenes, such as the full-length ∼15 kb dystrophin transgene.^25^ The downside of lentiviruses is that the retrotranscribed DNA genome is integrated into the host genome. Insertion of the transgene is random thus depending on the location it may cause mutations as well as deactivation or activation of tumor suppressor or oncogenes, respectively.^26,27^ In this study, we discuss two retinal diseases where lentiviruses are used in clinical trials to deliver the therapeutic transgenes.

### PR and RPE diseases and related genes

#### Stargardt's disease

Stargardt's disease (STGD) is an IRD that causes juvenile macular degeneration leading to blindness at a young age. There are four different forms of STGD (1–4) and three known disease genes.^28,29^ STGD1 is autosomal recessive and caused by mutations in the PR-specific ATP-binding cassette (ABC) transporter gene, *ABCA4*, which is a key protein of the visual cycle that transports N-retinylidene-phosphatidylethanolamine (N-ret-PE) to the cytosolic side of the PR disc membrane.^30^ Mutations in *ABCA4* result in misfolded or dysfunctional proteins that lead to inefficient transportation of N-ret-PE and consequently N-ret-PE condensation. This ultimately causes lipofuscin accumulation in the RPE, followed by RPE atrophy and retinal degeneration.^31–33^

In June 2011, a phase I/II clinical trial (NCT01367444) sponsored by Sanofi used a recombinant lentiviral vector based on the equine infectious anemia virus to express the full-length *ABCA4* gene (SAR422459) as a therapy to treat STGD1 patients. Because of the size of the *ABCA4* gene, the lentivirus was the preferred therapeutic vector. The study used a subretinal injection to target PR cells in 27 patients who were divided into 7 cohorts with a total of three different doses: 1.8 × 10^5^ transducing units (TU), 6 × 10^5^ TU, and 1.8 × 10^6^ TU. Three patients showed serious adverse events, including increased intraocular pressure (1.8 × 10^5^ TU), uveitis (1.8 × 10^6^ TU), and chorioretinopathy (1.8 × 10^6^ TU). In preclinical studies with mice, rabbits, and nonhuman primates, there were no serious adverse events besides some transient mild inflammation.^34,35^

The study was terminated in 2012 with outcomes established at 48 weeks postinjection. Patients have since been enrolled in a 15-year long-term study to follow-up on adverse events, best-corrected visual acuity (BCVA) and retinal degeneration (NCT01736592). A 3-year follow-up safety study published in March 2022 with 22 out of the original 27 patients reported that, of the 22 patients, all experienced at least one adverse event.^36^ Although most events were related to the surgical procedure, ∼10% of the events were caused by the interventional drug SAR422459 itself. No patient showed significant improvement in visual function. More investigations are needed to understand the safety and efficacy of the current treatment regimen and design.^36^

### Mutation-independent approaches

#### Age-related macular degeneration

AMD is the leading cause of blindness in the United States. There are two advanced disease forms of AMD: the exudative nAMD and the dry form. Although only ∼20% of patients have nAMD, this form leads to a fast progression of vision loss if untreated. The standard of care for nAMD is to inhibit the function of the vascular endothelial growth factor (VEGF), a growth factor that regulates neovascularization.^37^ Monoclonal antibodies or fusion proteins against VEGF, such as ranibizumab, bevacizumab, and aflibercept, are current anti-VEGF drugs used to treat nAMD.^38^ However, these anti-VEGF drugs require repeat injections to maintain stable inhibition of VEGF, burdening patients, their families, and health care providers.^39^ Thus, the development of a viral vector that transduces retinal cells with an anti-VEGF transgene for endogenous anti-VEGF production is an ideal strategy to overcome the repeat injection problem.

In 2011, Oxford BioMedica started a phase I clinical trial that treated nAMD patients with RetinoStat (NCT01301443). RetinoStat is a lentiviral vector-based therapy that expresses angiostatin and endostatin, which are natural angiogenesis inhibitors.^40^ Patients (*n* = 21) were subretinally injected with 2.4 × 10^4^, 2.4 × 10^5^, or 8 × 10^5^ TU to determine the long-term treatment efficacy. There was no dose-related toxicity in all the groups. Transgene expressions peaked between 12 and 24 weeks and levels remained stable for 2.5 years.^40^ Neovascular areas, however, showed no improvement and visual outcome, as measured by BCVA, only showed improvement in the subset of patients who received a high dose of RetinoStat. Only one patient developed a procedure-related adverse event (retinal hole). A 15-year follow-up study is underway to determine the long-term safety of this lentiviral anti-VEGF treatment (NCT01678872).

## ADENO-ASSOCIATED VIRUS

Recombinant adeno-associated-viruses (rAAVs) are small single-stranded DNA viruses with a viral genome of ∼4.7 kb. The transgene is flanked on either end by inverted terminal repeats, which are the only sequences of the viral genome that are used for gene therapy purposes. Gene expression from rAAV vectors persists for decades although the viral genome does not integrate into the host genome. It remains in the nucleus as episomal monomeric and/or concatemeric circles. This makes rAAV an ideal vector for postmitotic cells.^41^ Although mitotic cells express the transgene after rAAV infection, expression levels can decline over time as the genome copies are slowly diluted with each cell division. To date, there are more than 100 AAV serotypes, many of which infect neurons.^42^ AAV serotypes, AAV2, AAV5, AAV7, AAV8, AAV9, and AAV.rh10, have been shown to efficiently transduce PRs following subretinal delivery.^43–45^

Interestingly, PR transduction efficiency in mouse correlates with rod PR outer and inner segment development.^45^ An *in vivo*-directed evolution study engineered a new AAV2 variant, AAV2.7m8, that delivers the transgene to the outer retina, including PRs, by the less harmful intravitreal injection technique.^6^ Transgenes delivered by this serotype have been shown to rescue PR-related disease phenotypes in animal models of LCA and retinoschisis, as well as being able to treat choroidal neovascularization (CNV) in a laser damage model of CNV.^6,46,47^ Currently, there are many clinical trials that use rAAV vectors to treat retinal disease. The following is a summary of these clinical trials.

### PR and RPE diseases and related genes

#### Achromatopsia

Achromatopsia is an autosomal recessive heterogenous CD that is caused by mutations in different genes responsible for cone PR function.^48,49^ This differs from normal color blindness, where only one of the three cone types is affected due to a mutation in one of the cone-specific opsins. Most achromatopsia mutations progress slowly, leaving ample time for therapeutic intervention. However, achromatopsia can manifest in patients as young as 6 months of age. The disease is characterized by lack of color vision followed by symptoms such as photophobia, nystagmus, and low visual acuity.^49^ Multiple genes related to achromatopsia have been identified,^49^ of these genes, the cyclic nucleotide-gated channel subunit beta 3 (*CNGB3*) is the most prevalent one followed by *CNGA3*, for which more than 40 different pathological mutations have been identified.^50–52^

Several companies have initiated clinical trials to examine the safety and efficacy of rAAV gene therapies to treat patients with mutations in *CNGB3* and *CNGA3* by delivering a normal copy of the transgene to PRs.

In 2015, research groups at the University Hospital Tübingen and the Ludwig Maximilian University of Munich started a *CNGA3* gene therapy clinical trial (NCT02610582). The gene therapy treatment, rAAV.hCNGA3, used the AAV8 serotype as capsid, the cone-specific arrestin-3 (*ARR3*) promoter, and the human *CNGA3* complementary DNA to transduce cones by subretinal injection.^53^ Three different doses were used, 1 × 10^10^, 5 × 10^10^, and 1 × 10^11^ vector genomes (vg) on a total of nine adult patients with a study follow-up period of 12 months.^53^ No adverse events were reported besides two patients who developed mild immune responses to the drug that were resolved with corticosteroids.^53^

The 1-year clinical evaluation revealed that patients had improved overall cone function including visual acuity and contrast sensitivity, paving the way for an achromatopsia gene therapy at a younger age. This is particularly important because lack of cone PR input at a young age can have long-term effects on the development of the visual cortex, limiting the benefits of the gene therapy in adult patients.^54^

In addition to this trial, two other companies, MeiraGTx and Applied Genetic Technologies Corp (AGTC), started separate phase I/II clinical trials to examine their AAV therapies in both children and adult patients with *CNGB3* and *CNGA3* achromatopsia (MeiraGTx: NCT03001310, NCT03758404; AGTC: NCT02935517, NCT02599922). AGTC examined two AAV2 gene therapy drugs targeting the genes *CNGA3* (AGTC-402) and *CNGB3* (AGTC-401). Patients were subretinally injected up to 300 μL of viral concentrations ranging from 4 × 10^10^ to 3.2 × 10^12^ vg/mL. The interim safety evaluation presented by AGTC reported some toxicity at the highest dose (3.2 × 10^12^ vg/mL) in children, but not in adults (AGTC website). Excitingly, the current data suggest that the AGTC-401 treatment improved photosensitivity in some patients. The effect of AGTC-402 appears less encouraging (AGTC website). Results from the MeiraGTx trials have not been posted yet.

In summary, the current outcomes from these clinical trials provide hopes for achromatopsia patients to have their cone function restored in the future.

#### X-linked RP

X-linked RP (XLRP) is caused by several genes with the *Retinitis Pigmentosa GTPase Regulator* gene (*RPGR*) accounting for 70% of all XLRP cases and around 11% of all RP^55^ cases. *RPGR* encodes for a protein that is located in rod PR outer segments and is required for proper rod function. Thus, the disease manifests as an X-linked RCD. The first phase I/II clinical study for XLRP was conducted by NightstaRx Ltd in 2017 (NCT03116113, XIRIUS). A codon-optimized AAV8-*RPGR* was subretinally injected into 18 patients in a dose escalation study. A 6-month phase I follow-up study found no dose-limiting toxicity, although mild retinal inflammation treatable by oral corticosteroids was observed at higher doses. Some patients demonstrated a reversal of vision loss associated with visual activity gains.^56^ MeiraGTx conducted a similar phase I/II (MGT009) dose escalation study to examine treatment with their AAV5-*RPGR* vector in XLRP patients (NCT03252847).

A recent presentation at the annual ARVO meeting (2022) reported an improvement in retinal sensitivity after 12 months of treatment (ARVO oral presentation). A phase III clinical trial with bilateral administration at two doses (2 × 10^11^, 4 × 10^11^ vg) and a follow-up study are underway (NCT04671433, NCT04794101).

In 2018, AGTC conducted a phase I/II clinical trial with 29 XLRP patients receiving subretinal injection of rAAV2tYF-GRK1-*RPGR* (AGTC-501; GRK1: G-coupled receptor kinase 1 promoter for PR-specific expression; NCT03316560). Patients were distributed in 5 different groups, with 21 patients receiving central macular injection and 8 patients receiving injections in the peripheral regions. Surgery-related mild to moderated adverse events were observed. In the 12- and 18-month follow-up examinations by optical coherence tomography, there was a significant improvement in the visible foveal ellipsoid zone that was seen at 12 months and sustained at 18 months in patients with a visible foveal ellipsoid zone at baseline (AGTC website). New phase II/III clinical trials to evaluate the efficacy, safety, and tolerability of AGTC-501 are now underway (NCT04850118). While all these *RPGR* clinical trials use subretinal injections for viral delivery, 4D Molecular Therapeutics uses intravitreal injections.

In October 2021, 4D Molecular Therapeutics released interim results of its phase I/II clinical trial using their AAV vector, R100, in advanced RPGR patients who have limited or no measurable PR region remaining and low or no retinal sensitivity (NCT04517149). No dose-limiting toxicity or serious adverse events were observed. Interestingly, two patients at their follow up examination (6 and 9 months) had an increase in retinal sensitivity in the treated eyes (News releases). In all, the data from *RPGR* clinical trials suggest that a therapy may become soon available to patients who suffer from mutations in this gene.

#### Phosphodiesterase 6

Phosphodiesterase 6 (PDE6) is a heterotetrameric complex that hydrolyzes cGMP and reduces its concentration in rod PRs in response to light activation of the G-protein-coupled receptor during phototransduction. The PDE6 complex is composed of an α, a β, and two γ subunits, each of which is important for PR function.^57^ Mutations in *PDE6* are autosomal recessive, and mutations in the α and β subunit each account for around 4% of all RP cases.^58,59^ The disease progresses as an RCD. Based on preclinical data,^60,61^ Coave Therapeutics has started a phase I/II study with their vector, AAV5-*hPDE6B*, using three different doses (NCT03328130), while STZ eyetrial with the University of Tübingen is conducting a phase I/II study with their vector, rAAV-*hPDE6A*, to evaluate the safety and efficacy in patients with PDE6A mutations (NCT04611503, PIGMENT). Both trials use the subretinal delivery route and are still ongoing.

#### MERTK

***MERTK*** is an RP-related gene that encodes a protein expressed in RPE cells. It is involved in the PR outer segment uptake by RPE cells.^62^ Although mutations in *MERTK* do not directly affect PR function, they cause PR degeneration by accumulation of PR outer segment debris in the subretinal space. The King Khaled Eye Specialist Hospital conducted a phase I study using subretinal injections to deliver rAAV2-VMD2-*hMERTK* into six patients (VMD2: vitelliform macular dystrophy 2 promoter which is RPE specific; NCT01482195). Several complications were observed; however, no severe adverse events were reported. Three patients had improved visual acuity after surgery; however, two of the three patients had lost improvement at the 2-year follow-up.^63^

Further studies are needed to investigate the safety and efficacy of this RP-related gene augmentation therapy. Interestingly, the FDA-approved gene therapy with Luxturna, which treats a different RPE-expressed gene (*RPE65*) that causes LCA2, while effective at improving vision has also shown problems with preventing PR death over time.^64^ It is possible that RPE-related gene therapies are more difficult to fine-tune. Lessons from the ongoing LCA2 trials^64^ and this trial will certainly help to improve gene therapies for the RPE-related genes.

#### Retinaldehyde-binding protein 1

Retinaldehyde-binding protein 1 (RLBP1) is an autosomal recessive RP-related gene, encoding RLBP1, a protein that is expressed by Müller glial and RPE cells.^65^ RLBP1 participates in the regeneration of the visual chromophore and is part of the visual cycle proteins. Its expression in the RPE is important for the visual cycle between the RPE and PRs, while its expression in Müller glial cells may help with the alternative visual cycle between cones and Müller glia.^66^ Novartis Pharmaceutical has started a phase I/II clinical trial with its drug, AAV8-*RLBP1* (CPK850). The trial plans to use five different doses that are delivered subretinal to examine the safety, tolerability, and efficacy of the drug (NCT03374657). No data are currently available for this trial.

#### Choroideremia

Choroideremia is a chorioretinal dystrophy leading to progressive vision loss.^67^ The disease is X-linked and caused by mutations in the *CHM* gene that encodes the Rab escort protein 1 (REP1), which is involved in intracellular trafficking.^68–70^ The exact disease mechanism remains unclear; however, it is believed that RPE cells are the first cells affected, followed by an RCD and choroid atrophy.^67,71–73^ There are >200 disease-related mutations that have been identified.^67^ Because most mutations result in loss of function of REP1, gene augmentation therapy has been the approach chosen to treat this chorioretinal degeneration.^74^

In 2011, the University of Oxford conducted the first phase I/II choroideremia AAV gene therapy trial (NCT01461213), utilizing the AAV2 vector, the chicken β-actin ubiquitous promoter, and the woodchuck hepatitis virus posttranscriptional regulatory element inserted downstream of the *CHM* transgene cassette to increase transgene expression in the retina.^75^ A total of six patients received subfoveal injections of 1 × 10^10^ vg (0.1 mL), with the exception of one patient who was injected at the lower dose (6 × 10^9^ vg) due to surgical complications.^76^ Early improvement was seen in two patients who were most advanced in their disease, while sustained improvement was seen in most of the other patients in a follow-up study at 3.5 years when compared with the untreated eye that showed progressive retinal degeneration.^76,77^

The patient who received the lower dose due to a concern of retinal stretch during the procedure had continued deterioration in visual acuity likely caused by progressive retinal degeneration.

Following this study, the same treatment was used in three additional phase I/II clinical trials (NCT02077361, University of Alberta; NCT02553135, University of Miami; and NCT02671539, University Hospital Tübingen, STZ eyetrial). In these clinical trials, a higher dose of the drug (1 × 10^11^ vg in 0.1 mL) was delivered subfoveal with varying outcomes between the tials.^78–80^ Most patients had a mild improvement in BCVA, with few patients showing a decrease in the Early Treatment Diabetic Retinopathy Study (ETDRS) letter scoring. Interestingly, two patients from different trials showed improved BCVA in the untreated eyes.^78,79^ Two patients from the University of Miami trial (NCT02553135) developed retinal holes at 24 months,^79^ likely due to the subfoveal injection into patients with preexisting disease-related retinal thinning. One patient from the University of Alberta trial (NCT02077361) developed intraretinal inflammation resulting in declined visual function.^78^

Since these initial trials, NightstaRx Ltd (A Biogen Company) started phase II and phase III clinical trials using AAV2 to deliver the *CHM* transgene (BIIB111). The phase II (NCT03507686, GEMINI) clinical trial examines the safety of bilateral sequential injections, while the phase III trial (NCT03496012, STAR) examines the outcomes of a unilateral one-time injection with a high and a low viral dose. In June 2021, Biogen announced that the results of the STAR study did not meet its primary endpoint of >15 ETDRS letters of improvement in treated patients (Biogen News Release).

Similarly, a sponsored safety and dose escalation phase I/II study by Spark Therapeutics with AAV2-hCHM (NCT02341807) showed no improvement in visual acuity in both the high-dose (1 × 10^11^ vg) and low-dose groups (5 × 10^10^ vg) at a 2-year follow-up examination^81^; however, longer periods of examination are needed for both the Biogen and the Sparks trial to evaluate the full therapeutic potential of these treatments.

Meanwhile, in 2020, 4D Molecular Therapeutics started the first phase I clinical trial to treat choroideremia by an intravitreal injection with a new capsid variant (NCT04483440). If this capsid reaches the outer retina efficiently, the approach would reduce the risk of retinal complications from the procedure as it avoids the more risky subretinal injections in a disease-related thin retina. The trial used two doses (3 × 10^11^, 1 × 10^12^ vg), and while results are not available yet, no serious adverse event was reported from the initial injections. In summary, while primary endpoints have often not been achieved in the choroideremia clinical trials, the partial improvements in BCVA and the reduction in disease progression seen in some patients bode well for the therapeutic future of this disease.

#### Leber's congenital amaurosis

LCA is an early-onset retinal dystrophy leading to blindness or severe visual impairment in infants.^82^ LCA is a heterogenous retinal disease family with more than 400 mutations in 14 LCA-associated genes, several of which are also implicated in other retinal diseases, such as RP.^83^ Luxturna, the first FDA-approved *in vivo* gene therapy for the eye, targets autosomal recessive LCA2 that is caused by mutations in *RPE65*.^84^ Because this disease gene has already an FDA-approved drug on the market and clinical trial results have been reviewed recently,^64^ we are not reviewing any LCA2-associated clinical trial here.

In addition to *RPE65*, *CEP290* (LCA10) is an LCA gene that accounts for ∼20% of LCA patients, making it one of the most frequent causes of LCA.^85^ The gene encodes a ciliary protein that is required to maintain PR structure and functions.^86^ Because *CEP290* is ∼8 kb in size, the limited packaging capacity of rAVV makes it difficult to develop a gene augmentation approach. In 2019, Iveric Bio announced a minigene program to treat LCA10 (IVERIC Bio), with a truncated *CEP290* transgene that could be packaged into AAV vectors and was shown to rescue PR function and morphology in a mouse model of *CEP290* through subretinal injection.^87^ The data provide a new path for minigene therapies with AAV vectors to rescue diseases caused by mutations in large genes.

A different approach to this problem is being developed by Editas Medicine. Clustered regularly interspaced short palindromic repeat DNA sequences (CRISPR) and Cas9 (CRISPR/Cas9) has gained popularity as a genetic editing tool in recent years. In 2020, two chemists, Emmanuelle Charpentier and Jennifer Doudna, were awarded the Noble Prize in chemistry for their pioneer work on CRISPR/Cas9. CRISPR/Cas9 consists of two components, the Cas9 enzyme and the guide RNA (gRNA) sequence, which together form the ribonucleoprotein (RNP) complex. The gRNA guides the RNP to a specific location by sequence homology, while the Cas9 enzyme generates double-strand breaks (DSBs). These DSBs can be repaired by nonhomology end joining, which results in gene insertions or deletions or by homology direct repair with an additional DNA template to allow for precise sequence insertion/replacement.^88^

Editas Medicine is now conducting the first RNP *in vivo* clinical trial in the eye (NCT03872479, BRILLIANCE) to treat LCA10 patients with intronic mutations in *CEP290* that lead to splicing defects. The drug, EDIT-101, uses AAV5 as a vector to package two gRNAs and Cas9. Expression of the transgene for Cas9 is restricted to PRs with the hGRK1 promoter. Adult and pediatric participants received a single subretinal injection of low (6 × 10^11^ vg/mL), medium (1.1 × 10^12^ vg/mL), or high (3 × 10^12^ vg/mL) concentration of virus. In the interim report, no severe adverse event or dose-limiting toxicity was observed with all the doses.

Early clinical benefits were observed at the medium dose (Editas Medicine website). Further clinical updates are expected later in 2022. In addition to EDIT-101, Editas Medicine is also in the process of examining two CRISPR/Cas9 drugs for other IRDs, EDIT-103 and EDIT-102. EDIT-103 has been developed to treat any mutations in the rhodopsin (*RHO)* gene that cause autosomal dominant RP. The strategy uses a dual AAV system to remove endogenous *RHO* (both normal and mutated alleles) and insert a codon-optimized *RHO* sequence, which is resistant to Cas9 cleavage, to restore functional RHO expressions. Preclinical trial experiments conducted in nonhuman primates showed a nearly ∼100% editing efficiency and restoration of PR morphology (ASGCT 2022). The other CRISPR editing drug, EDIT-102, is to target Usher syndrome (USH2A) by deleting exon 13.

If successful, these approaches provide an alternative gene modification technique to treat IRDs. In particular, dominant mutations or mutations in genes that are too large to package in an rAAV vector will benefit from this approach.

### INL and GCL diseases

#### X-linked retinoschisis

X-linked retinoschisis (XLRS) is a leading cause of inherited juvenile macular degeneration in males.^89,90^ Affected males show visual impairment and bilateral foveal schisis early in life, generally between infancy and school age, however, the level of visual impairment varies.^91,92^ The disease is an X-linked disorder that is caused by mutations in the *XLRS1* gene. *XLRS1* encodes retinoschisin 1 (RS1), a cell-surface adhesion protein expressed in PRs and bipolar cells that plays a role in cell–cell interaction causing a splitting (schisis) of retinal layers when mutated.^93,94^ In 2015, the National Eye Institute (NEI) sponsored the first XLRS gene therapy phase I/IIa clinical trial (NCT02317887), using rAAV8 to deliver *RS1* into patients through intravitreal injection.

At an 18-month follow-up, no significant changes in visual acuity were observed in treated versus untreated eyes, however, one patient injected with the high dose (1 × 10^11^ vg vs. 1 × 10^10^ vg) showed a transient closure of macular cavities at 2 weeks postinjection that was most likely due to RS1 protein function.^95^ AGTC initiated a different phase I/II trial (NCT02416622) that used AAV2 (rAAV2tYF-CB-*hRS1*) to transduce retinal cells through intravitreal injection. Unfortunately, the 6-month evaluation showed no improvement in visual activity, in both adult and pediatric patients, leading to the decision to terminate further production of the product (AGTC news release, December 2018). The ambiguity in clinical outcomes observed in these studies may be due to insufficient transduction of PRs by both vectors, leading to RS1 expression below therapeutic levels.

This problem may be overcome by the clinical program announced by Atsena Therapeutics. The company is planning an XLRS gene therapy program using a new AAV capsid, AAV-SPR, which spreads laterally beyond the injection site after subretinal injection (Atsena Therapeutics news release October 18, 2021). Subretinal injection will target many more PRs. Preclinical studies in NHPs show an expanded transduction profile, and studies in mouse with AAV.RSP-*RP1* show a dose-dependent improvement in retinal structure and function (ARVO presentation, 2022).

#### Leber hereditary optic neuropathy

LHON is a mitochondrial disorder caused by pathogenic mutation in mitochondrial DNA (mtDNA), which is passed on by maternal transmission.^96,97^ The disease affects primarily retinal ganglion cells (RGCs), which are the output neurons that bundle their axons at optic disc to form the optic nerve that exits the eye. The long RGCs axons are composed of unmyelinated segments in the intraocular portion and become myelinated once they exit the ocular globe.^98^ Why these mutations affect primarily RGCs remains unclear. The severity of LHON varies between no symptoms to vision loss,^98–100^ which is caused by a gradual degeneration of the retinal nerve fiber layer resulting in optic nerve atrophy.^100,101^ The first pathogenic mutation identified is in the *ND4* gene, a subunit of Complex I, which functions in the mitochondrial respiratory chain.^102–104^

Gene therapies for LHON require allotopic expression to restore mitochondrial function. This is achieved by adding a mitochondrial targeting sequence to the *ND4* transgene. A proof of concept that allotropic expression can restore mitochondrial function was shown in cybrid cells containing mutant mitochondria.^102^ This experiment paved the way for several clinical trials.

A phase I trial conducted by the Bascom Palmer Institute at University of Miami treated initially five LHON blind participants unilaterally with intravitreal injection of a self-complementary AAV2 (scAAV-P1*ND4*v2) (NCT02161380). Two of the initial five patients showed improvement in visual acuity at 6 months. In a second round with 14 patients, 6 patients with bilateral visual loss <12 months showed significant difference in visual acuity between the treated and fellow eye after a single intravitreal injection of the therapeutic drug. No severe adverse events were observed.^105,106^

GenSight biologic also started a phase I/II clinical trial to examine their gene therapy, rAAV2-*ND4* (GS010; Lumevoq: lenadogene nolparvovec; NCT02064569). Mild adverse events were reported including intraocular inflammation as well as anterior chamber inflammation.^107,108^ Following this trial, two phase III clinical studies, RESCUE and REVERSE, evaluated the efficacy and safety of unilateral intravitreal injection of rAAV2-*ND4* into ND4-LHON patients who had vision loss <6 months (NCT02652767, RESCUE) or between 6 and 12 months (NCT02652780, REVERSE). Both studies together with an additional long-term follow-up study (NCT03406104, RESTORE) show sustained improvement in BCVA up to 4 years after treatment in both treated and fellow eyes, suggesting a contralateral effect of Lumevoq.^109–111^ The drug is expected to be reviewed by the Committee for Medicinal Products for Human use (CHMP) at the end of 2022 and anticipated to have commercial launch in 2023.

In addition, GenSight has started a phase III clinical trial to measure the efficacy and safety of bilateral intravitreal injection of Lumevoq (REFLECT NCT03293524).

A phase II//III clinical trial done by Huazhong University of Science and Technology treated nine patients with rAAV2-*ND4* by intravitreal injection with doses ranging from 5 × 10^9^ to 1 × 10^10^ vg (NCT01267422). Six patients showed improved visual acuity and no patient showed serious adverse events by 9 months post-treatment.^112^ Visual acuity improvement remained stable at 36 months in five of the patients and a similar contralateral effect of the treatment was observed in some patients. The study also reported a patient who received a bilateral injection, with the second injection 12 months after the first eye was treated. The patient showed decreased visual acuity in the first eye after the second eye was treated. Intraocular immune response to AAV2 is a potential explanation; however, this patient showed a normal humoral immune response before the second injection.^113^

Thus, further studies are needed to elucidate the cause of declining visual acuity after the second contralateral injection. In all, the data obtained from ongoing clinical trials for LHON suggest that mutations in this disease gene may soon become treatable with an approved drug.

### Mutation-independent approaches

#### Optogenetic

Optogenetic therapy is an approach that restores visual sensitivity by introducing light sensors (opsins) into light insensitive cells such as ganglion cells when PRs are degenerated in the retina.^114,115^ The approach is being tested in the advanced disease stages of RP, where PRs have either completely died off or become dysfunctional. In animal models, introducing a microbial rhodopsin, channelrhodopsins (ChRs), or an ambient light activatable multicharacteristic opsin (MCO1) improves visual guided behaviors and light responses in retinas that lack PRs.^114–117^ There are currently four clinical trials that use an optogenetic approach to restore vision in RP patients. AbbVie (Allergan) sponsored a phase I/II clinical trial that uses the AAV2 vector to transfer *Channelrhodopsin-2* (*ChR2*) (RST-001) to RGCs by intravitreal injection (NCT02556736).

GenSight Biologics sponsored a phase I/II study (NCT03326336, PIONEER) that combines optogenetic therapy after intravitreal injection of rAAV2.7m8-CAG-*ChrimsonR-tdTomato* (GS030) with light stimulating goggles to recover visual function. Excitingly, one patient has been reported to be able to perceive objects and has object sensation activity in the visual cortex after receiving this therapy.^118^ NanoScope Therapeutics, Inc. is conducting a phase I/II clinical trial (NCT04919473), and a separate phase II study (NCT04945772, RESTORE), to examine the safety and efficacy of intravitreal injection of AAV2-*MCO* (MCO-010). Besides the success with one treated patient in the GenSight trial, there are currently limited data available for all other trials. However, restoring some sense of vision in a blind person gives hope to many that suffer from RP and other blinding diseases.

#### rAAV-mediated anti-VEGF

rAAV-mediated anti-VEGF clinical trials are being conducted for neovascular eye pathologies in diabetic macular edema (DME), diabetic retinopathy, and nAMD. DME and diabetic retinopathy are both multifactorial diseases that are associated with uncontrolled hyperglycemia in diabetic patients.^119^ The constant hyperglycemic condition results in abnormal metabolic activity that may increase retinal inflammation resulting in neurodegeneration and disruption of the blood–retinal barriers, promoting diabetic edemas.^120^ Anti-VEGF drugs are one class of the drugs used to treat retinal edema and prevent the breakdown of the blood–retinal barriers. Adverum Biotechnology had a clinical study (NCT04418427, INFINITY) to use AAV2.7m8 to intravitreally deliver an anti-VEGF transgene, aflibercept (ADVM-022), into DME patients. The study examined the safety and efficacy of ADVM-022 in DME patients at two doses (2 × 10^11^ and 6 × 10^11^ vg/eye).

In April 2021, Adverum reported a severe adverse reaction of hypotony in one patient, and since then, additional patients who were treated with the higher dose experienced adverse events. None of the 13 DME patients treated with the lower dose experienced any adverse events. Nonetheless, the company has decided to not continue the ADVM-022 program for DME (Adverum news release, July 2021). Around the same time as the INFINITY trial, Adverum also started a clinical trial with the same drug (ADVM-022) to treat intravitreally nAMD patients (NCT03748784, OPTIC). In contrast to DME patients, no dose-limiting toxicity was seen in nAMD patients. A single intravitreal injection of the low dose of ADV-022 has been shown to have long-duration efficacy (3 years) with no occurrence of inflammation or any adverse events (Adverum news, June 2022).

In addition to Adverum, four other groups are also conducting clinical trials on rAAV-mediated anti-VEGF drug delivery. The Lions Eye Institute in collaboration with Adverum (NCT01494805), and separately, Genzyme (NCT01024998), made rAAV-based drugs that deliver transgenes for the soluble form of the VEGF receptor (sFLT-1). Reports from the phase I/II clinical trial conducted by the Lions Eye Institute showed that subretinal injection of rAAV2-*sFLT-1* (1 × 10^11^ vg), along with intravitreal injections of ranibizumab, is safe and remains stable for more than 3 years.^121–124^

In the Genzyme-sponsored phase I clinical trial (NCT01024998), 19 patients were treated with a single intravitreal injection with 1 of the following 4 doses: 2 × 10^8^, 2 × 10^9^, 6 × 10^9^, and 2 × 10^10^ vg. The 1-year follow-up study showed that two patients in the high-dose cohort exhibited drug-related adverse events. Eleven of 19 (57%) patients had reversible intraretinal or subretinal fluids from baseline, and six patients showed reduction in fluids and improved vision.^125^

4D Molecular Therapeutics has started a phase I/II clinical trial to deliver their rAAV anti-VEGF drug, 4D-150, through intravitreal injections (NCT05197270). The drug uses a microRNA that targets VEGF-C and a codon optimized sequence of aflibercept. No data are available for this trial yet. Finally, Regenxbio, Inc. uses an AAV8-based anti-VEGF drug (RGX-314), which is an antibody-based anti-VEGF drug, through subretinal injections into nAMD patients. RGX-314 has already been through phase I and II studies (NCT03066258, NCT04832724, NCT03999801, NCT04704921) and is currently undergoing the ASCENT phase III pivotal 2 study (NCT05407636; ASCENT). The initial trial consisted of a five-dose (3 × 10^9^, 1 × 10^10^, 6 × 10^10^, 1.6 × 10^11^, and 2.5 × 10^11^ vg) escalation study on 42 patients.

Remarkably, patients in cohorts 3–5 had an ∼60–84% reduction in follow-up injections of the regular anti-VEGF drug after receiving RGX-314. The drug appears to be well tolerated, with only one patient showing a significant decrease in vision at the highest dose.

Regenxbio, Inc. is also conducting two clinical trials (NCT04567550 ALTITUDE; NCT04514653 AAVIATE) for their drug (RGX-314) with the newly developed less invasive suprachoroidal injection procedure.^7^ Preclinical studies showed that suprachoroidal injections of RGX-314 provide the same level of inhibition of vascular leakage when compared with subretinal injections.^8^ The phase II clinical trials deliver the gene therapy product RGX-314 into patients with diabetic retinopathy and nAMD, respectively. In the first cohort, diabetic retinopathy patients were treated with 2.5 × 10^11^ vg. No serious drug-related adverse events have been reported during the 3 months of the study and some patients show improvements in their vision. Similar results are reported in the nAMD group, with no serious adverse events in patients treated with 2.5 × 10^11^ and 5 × 10^11^ vg of RGX-314. At a 6-month follow-up, patients treated with the 2.5 × 10^11^ vg dose show stable visual acuity and retinal thickness.

The data, while yet limited, are encouraging, especially because this delivery method, if effective at targeting PRs in humans, would significantly reduce the risks associated with subretinal injections for many of the IRDs.

### Geographic atrophy

Geographic atrophy (GA) is an advanced form of AMD that affects ∼80% of all advanced AMD patients making it the main cause for visual impairment in the elderly of the industrialized world.^126^ GA is also often referred to as dry AMD. It is characterized by geographic loss of RPE and PR cells, resulting in severe vision loss once the macular region is affected. The clinical sign preceding GA is the buildup of drusen, which are yellow, lipid-rich sub-RPE deposits that accumulate around the macular region. Among the nongenetic risk factors, age, smoking, and diet are the most relevant factors,^127–129^ while genetic risk factors congregate around genetic variants of the complement system, genes related to lipid metabolism, and extracellular matrix proteins.^130^ Despite the identification of more than 50 risk genes,^130^ and a new disease model that implicates PRs in the pathogenesis,^131^ the underlying cause for AMD remains unclear.

Thus, there is currently no treatment to prevent the onset or progression of GA. However, given the prevalence of dry AMD and the unmet needs to find a treatment due to the increase in the aging population, there are many (∼30) registered clinical trials for dry AMD.^132^ Most of these trials focus on known risk factors, with two of them using an rAAV2 gene therapy to target the complement system. Several genetic variants of the complement system have been associated with AMD, suggesting a critical role of complement system in disease progression.^133^ In addition, drusen, which precede GA, have been proposed to trigger an inflammatory response that is believed to contribute to disease progression.^134^

The first dry AMD gene therapy clinical trial (NCT03144999, HMR-001) was initiated by Hemera Biosciences and then later transferred to Janssen Research. The trial uses AAV2 to deliver by intravitreal injection a transgene that expresses from the ubiquitous CAG promoter the soluble form of CD59 (HMR59), which is a protein that inhibits the formation of the membrane attack complex.^135^ Three doses were used in a dose escalation trial to inject 17 patients with GA. Four eyes developed a mild inflammation, two of which were treated with topical steroids, while in the other two the inflammation resolved during observations. Most patients in the high-dose cohort showed a reduced growth rate of the GA lesion (9 out of 11), while two who developed an inflammation had an accelerated growth in the GA lesion.

None of the patients had yet converted to nAMD.^136^ The same drug is now being tested in 24 patients with nAMD with additional anti-VEGF injections as needed (NCT03585556). Two doses were used (3.56 × 10^11^, 1.07 × 10^12^ vg) with an anti-VEGF pretreatment 7 days before the viral delivery, and a 7-day dose of oral corticosteroids after the viral delivery. Despite the oral steroids, three patients developed a mild inflammation that required oral and/or topical treatments. Four out of 22 patients with 6-month follow-up data did not require any additional anti-VEGF injections.

In 2018, Gyroscope Therapeutics Limited started a phase I/II clinical trial (NCT03846193 FOCUS) to deliver complement factor I (FI), a complement factor that can inactivate the complement system, and for which several AMD-associated variants have been identified.^127^ Patients (31 thus far) received a single subretinal injection of AAV2 expressing FI (GT005) from the ubiquitous CAG promoter^137^ in the following dose escalation study: 2 × 10^10^, 5 × 10^10^, or 2 × 10^11^ vg. No serious adverse events were reported thus far, although mild events such as cataracts developed in nine cases. There was no drug-related inflammation, and the interim results show sustained increased expression of vitreous FI and a reduction in downstream proteins involved in overactivation of the complement system (Presentation).

In summary, all three trials are still ongoing and more data are need for both of these new rAAV drugs to determine their efficacy in delaying progression of dry AMD and conversion to nAMD. However, the preliminary data are encouraging, in particular for individuals with complement component risk alleles.

## CONCLUSIONS

The number of clinical trials for retinal gene therapies has increased dramatically over the last decade (see Supplementary Table S1 for a summary and link of trials listed in this review). We did not discuss gene therapy trials related to newer nonviral gene modification technologies such as gene modification by antisense oligonucleotides.^138^ The most promising trial results of the ones presented here are the ones for achromatopsia, XLRP (*RPGR* gene mutations), nAMD (inhibition of VEGF function), and LHON (*ND4* gene mutations). The LHON therapy, in particular, is very advanced and may become soon available if it is approved later this year by the CHMP. The data on the use of the CRISPR/Cas9 technology for LCA10 are also very encouraging. While the trial is still in its infancy, a positive outcome from this trial would open up many more disease mutations for treatment.

The advantage of this technology is that it could correct endogenous gene mutations. This would overcome the problem of having to fine tune a therapy to achieve the correct therapeutic expression levels of the transgene.

Inflammatory responses to the therapies discussed in this review have been a reoccurring theme. In most cases, these inflammatory reactions have been treated with topical or oral steroids. They tend to resolve with treatment and rarely pose a long-term risk. Interestingly, there seems to be little correlation between dose and inflammation with ocular gene therapy.^139,140^ Some of the inflammatory responses may also depend on the transgene, its biological function, and the disease state of the patient.^140^ For a more in-depth review on inflammatory responses seen in ocular gene therapies, we refer the readers to a recently published review on the topic.^139^

In summary, there is an FDA-approved drug for an RPE cell expressed gene (*RPE65*), potentially soon a new approved drug for a mutation that affects ganglion cells (LHON therapy), and promising data on rod and cone PR-specific gene mutations (*RPGR*, *CNGA3*, *CNGB3*). Thus, soon we may have at least one treatment for each of the cell types most likely affected by mutations that lead to blindness. The knowledge gained from the *RPGR* and *ND4* gene therapies will be instrumental in optimizing and improving the CRISPR/Cas9 therapies for PRs, and the optogenetic therapies for ganglion cells. The results of these trials give hope that vision loss can not only be prevented in the future, but maybe even restored in blind individuals who have no PRs left.

## Supplementary Material

Supplemental data

## References

[1] Wirth T, Parker N, Ylä-Herttuala S. History of gene therapy. Gene 2013;525(2):162–169; doi: 10.1016/j.gene.2013.03.137 23618815

[2] Wang D, Gao G. State-of-the-art human gene therapy: Part I. Gene delivery technologies. Discov Med 2014;18(97):67–77. 25091489 PMC4440413

[3] Daiger SP. Identifying retinal disease genes: How far have we come, how far do we have to go? Novartis Found Symp 2004;255:17–27; discussion 27–36, 177–178: doi:10.1002/0470092645.ch3 14750594 10.1002/0470092645.ch3 PMC2582379

[4] Streilein JW. Ocular immune privilege: Therapeutic opportunities from an experiment of nature. Nat Rev Immunol 2003;3(11):879–889; doi: 10.1038/nri1224 14668804

[5] Ramachandran PS, Lee V, Wei Z, et al. Evaluation of dose and safety of AAV7m8 and AAV8BP2 in the non-human primate retina. Hum Gene Ther 2017;28(2):154–167; doi: 10.1089/hum.2016.111 27750461 PMC5312498

[6] Dalkara D, Byrne LC, Klimczak RR, et al. In vivo-directed evolution of a new adeno-associated virus for therapeutic outer retinal gene delivery from the vitreous. Sci Transl Med 2013;5(189):189ra76; doi: 10.1126/scitranslmed.3005708 23761039

[7] Kansara V, Muya L, Wan C-R, et al. Suprachoroidal delivery of viral and nonviral gene therapy for retinal diseases. J Ocul Pharmacol Ther 2020;36(6):384–392; doi: 10.1089/jop.2019.0126 32255727 PMC7404827

[8] Ding K, Shen J, Hafiz Z, et al. AAV8-vectored suprachoroidal gene transfer produces widespread ocular transgene expression. J Clin Invest 2019;129(11):4901–4911; doi: 10.1172/JCI129085 31408444 PMC6819121

[9] Petit L, Khanna H, Punzo C. Advances in gene therapy for diseases of the eye. Hum Gene Ther 2016;27(8):563–579; doi: 10.1089/hum.2016.040 27178388 PMC4991575

[10] Punzo C, Kornacker K, Cepko CL. Stimulation of the insulin/mTOR pathway delays cone death in a mouse model of retinitis pigmentosa. Nat Neurosci 2009;12(1):44–52; doi: 10.1038/nn.2234 19060896 PMC3339764

[11] Venkatesh A, Ma S, Le YZ, et al. Activated mTORC1 promotes long-term cone survival in retinitis pigmentosa mice. J Clin Invest 2015;125(4):1446–1458; doi: 10.1172/JCI79766 25798619 PMC4396488

[12] Venkatesh A, Ma S, Punzo C. TSC but not PTEN loss in starving cones of retinitis pigmentosa mice leads to an autophagy defect and mTORC1 dissociation from the lysosome. Cell Death Dis 2016;7(6):e2279; doi: 10.1038/cddis.2016.182 PMC5108335 27362797

[13] Petit L, Ma S, Cipi J, et al. Aerobic glycolysis is essential for normal rod function and controls secondary cone death in retinitis pigmentosa. Cell Rep 2018;23(9):2629–2642; doi: 10.1016/j.celrep.2018.04.111 29847794 PMC5997286

[14] Stearns G, Evangelista M, Fadool JM, et al. A mutation in the cone-specific pde6 gene causes rapid cone photoreceptor degeneration in zebrafish. J Neurosci 2007;27(50):13866–13874; doi: 10.1523/JNEUROSCI.3136-07.2007 18077698 PMC6673616

[15] Kanow MA, Giarmarco MM, Jankowski CS, et al. Biochemical adaptations of the retina and retinal pigment epithelium support a metabolic ecosystem in the vertebrate eye. Elife 2017;6:e28899; doi: 10.7554/eLife.28899 PMC5617631 28901286

[16] Ferrari S, Di Iorio E, Barbaro V, et al. Retinitis pigmentosa: Genes and disease mechanisms. Curr Genomics 2011;12(4):238–249; doi: 10.2174/138920211795860107 22131869 PMC3131731

[17] Hartong DT, Berson EL, Dryja TP. Retinitis pigmentosa. Lancet 2006;368(9549):1795–1809; doi: 10.1016/S0140-S6736(06)69740-7 17113430

[18] Wingfield JL, Lechtreck K-F, Lorentzen E. Trafficking of ciliary membrane proteins by the intraflagellar transport/BBSome machinery. Essays Biochem 2018;62(6):753–763; doi: 10.1042/EBC20180030 30287585 PMC6737936

[19] Brancati F, Dallapiccola B, Valente EM. Joubert Syndrome and related disorders. Orphanet J Rare Dis 2010;5:20; doi: 10.1186/1750-1172-5-20 PMC2913941 20615230

[20] Ronquillo CC, Bernstein PS, Baehr W. Senior-Løken syndrome: A syndromic form of retinal dystrophy associated with nephronophthisis. Vision Res 2012;75:88–97; doi: 10.1016/j.visres.2012.07.003 22819833 PMC3504181

[21] Hartill V, Szymanska K, Sharif SM, et al. Meckel-Gruber syndrome: An update on diagnosis, clinical management, and research advances. Front Pediatr 2017;5:244; doi: 10.3389/fped.2017.00244 PMC5701918 29209597

[22] Mali S. Delivery systems for gene therapy. Indian J Hum Genet 2013;19(1):3–8; doi: 10.4103/0971-6866.112870 23901186 PMC3722627

[23] Sakuma T, Barry MA, Ikeda Y. Lentiviral vectors: Basic to translational. Biochem J 2012;443(3):603–618; doi: 10.1042/BJ20120146 22507128

[24] Balaggan KS, Ali RR. Ocular gene delivery using lentiviral vectors. Gene Ther 2012;19(2):145–153; doi: 10.1038/gt.2011.153 22052240

[25] Counsell JR, Asgarian Z, Meng J, et al. Erratum: Lentiviral vectors can be used for full-length dystrophin gene therapy. Sci Rep 2017;7:46880; doi: 10.1038/srep46880 PMC7365309 28849794

[26] Schlimgen R, Howard J, Wooley D, et al. Risks associated with lentiviral vector exposures and prevention strategies. J Occup Environ Med 2016;58(12):1159–1166; doi: 10.1097/JOM.0000000000000879 27930472 PMC5152689

[27] Romano G, Marino IR, Pentimalli F, et al. Insertional mutagenesis and development of malignancies induced by integrating gene delivery systems: Implications for the design of safer gene-based interventions in patients. Drug News Perspect 2009;22(4):185–196; doi: 10.1358/dnp.2009.22.4.1367704 19536363

[28] Allikmets R. A photoreceptor cell-specific ATP-binding transporter gene (ABCR) is mutated in recessive Stargardt macular dystrophy. Nat Genet 1997;17(1):122; doi:10.1038/ng0997-122a. 10.1038/ng0997-122a 9288113

[29] Zhang K, Kniazeva M, Han M, et al. A 5-bp deletion in ELOVL4 is associated with two related forms of autosomal dominant macular dystrophy. Nat Genet 2001;27(1):89–93; doi: 10.1038/83817 11138005

[30] Molday RS, Zhong M, Quazi F. The role of the photoreceptor ABC transporter ABCA4 in lipid transport and Stargardt macular degeneration. Biochim Biophys Acta 2009;1791(7):573–583; doi: 10.1016/j.bbalip.2009.02.004 19230850 PMC2746242

[31] Weng J, Mata NL, Azarian SM, et al. Insights into the function of Rim protein in photoreceptors and etiology of Stargardt's disease from the phenotype in abcr knockout mice. Cell 1999;98(1):13–23; doi: 10.1016/S0092-S8674(00)80602-9 10412977

[32] Mata NL, Weng J, Travis GH. Biosynthesis of a major lipofuscin fluorophore in mice and humans with ABCR-mediated retinal and macular degeneration. Proc Natl Acad Sci U S A 2000;97(13):7154–7159; doi: 10.1073/pnas.130110497 10852960 PMC16515

[33] Zhang N, Tsybovsky Y, Kolesnikov AV, et al. Protein misfolding and the pathogenesis of ABCA4-associated retinal degenerations. Hum Mol Genet 2015;24(11):3220–3237; doi: 10.1093/hmg/ddv073 25712131 PMC4424957

[34] Binley K, Widdowson P, Loader J, et al. Transduction of photoreceptors with equine infectious anemia virus lentiviral vectors: Safety and biodistribution of StarGen for Stargardt disease. Invest Ophthalmol Vis Sci 2013;54(6):4061–4071; doi: 10.1167/iovs.13-11871 23620430 PMC3681475

[35] Kong J, Kim S-R, Binley K, et al. Correction of the disease phenotype in the mouse model of Stargardt disease by lentiviral gene therapy. Gene Ther 2008;15(19):1311–1320; doi: 10.1038/gt.2008.78 18463687 PMC3110063

[36] Parker MA, Erker LR, Audo I, et al. Three-year safety results of SAR422459 (EIAV-ABCA4) gene therapy in patients with ABCA4-associated Stargardt disease: An open-label dose-escalation phase I/IIa clinical trial, cohorts 1–5. Am J Ophthalmol 2022;240:285–301; doi: 10.1016/j.ajo.2022.02.013 35248547 PMC9308722

[37] Gragoudas ES, Adamis AP, Cunningham ET Jr, et al. Pegaptanib for neovascular age-related macular degeneration. N Engl J Med 2004;351(27):2805–2816; doi: 10.1056/NEJMoa042760 15625332

[38] Kovach JL, Schwartz SG, Flynn HW Jr, et al. Anti-VEGF treatment strategies for wet AMD. J Ophthalmol 2012;2012:786870; doi: 10.1155/2012/786870 PMC3317200 22523653

[39] Baek SK, Kim JH, Kim JW, et al. Increase in the population of patients with neovascular age-related macular degeneration who underwent long-term active treatment. Sci Rep 2019;9(1):13264; doi: 10.1038/s41598-019-49749-y PMC6744448 31519960

[40] Campochiaro PA, Lauer AK, Sohn EH, et al. Lentiviral vector gene transfer of endostatin/angiostatin for macular degeneration (GEM) study. Hum Gene Ther 2017;28(1):99–111; doi: 10.1089/hum.2016.117 27710144 PMC5278797

[41] Naso MF, Tomkowicz B, Perry WL III, et al. Adeno-associated virus (AAV) as a vector for gene therapy. BioDrugs 2017;31(4):317–334; doi: 10.1007/s40259-017-0234-5 28669112 PMC5548848

[42] Castle MJ, Turunen HT, Vandenberghe LH, et al. Controlling AAV tropism in the nervous system with natural and engineered capsids. Methods Mol Biol 2016;1382:133–149; doi: 10.1007/978-1-4939-3271-9_10 26611584 PMC4993104

[43] Allocca M, Mussolino C, Garcia-Hoyos M, et al. Novel adeno-associated virus serotypes efficiently transduce murine photoreceptors. J Virol 2007;81(20):11372–11380; doi: 10.1128/JVI.01327-07 17699581 PMC2045569

[44] Pang J-J, Lauramore A, Deng W-T, et al. Comparative analysis of in vivo and in vitro AAV vector transduction in the neonatal mouse retina: Effects of serotype and site of administration. Vision Res 2008;48(3):377–385; doi: 10.1016/j.visres.2007.08.009 17950399

[45] Petit L, Ma S, Cheng S-Y, et al. Rod outer segment development influences AAV-mediated photoreceptor transduction after subretinal injection. Hum Gene Ther 2017;28(6):464–481; doi: 10.1089/hum.2017.020 28510482 PMC5488363

[46] Cheng S-Y, Luo Y, Malachi A, et al. Low-dose recombinant adeno-associated virus-mediated inhibition of vascular endothelial growth factor can treat neovascular pathologies without inducing retinal vasculitis. Hum Gene Ther 2021;32(13–14):649–666; doi: 10.1089/hum.2021.132 34182803 PMC8312021

[47] Grishanin R, Vuillemenot B, Sharma P, et al. Preclinical evaluation of ADVM-022, a novel gene therapy approach to treating wet age-related macular degeneration. Mol Ther 2019;27(1):118–129; doi: 10.1016/j.ymthe.2018.11.003 30528929 PMC6319194

[48] Hassall MM, Barnard AR, MacLaren RE. Gene therapy for color blindness. Yale J Biol Med 2017;90(4):543–551. 29259520 PMC5733843

[49] Remmer MH, Rastogi N, Ranka MP, et al. Achromatopsia: A review. Curr Opin Ophthalmol 2015;26(5):333–340; doi: 10.1097/ICU.0000000000000189 26196097

[50] Khan NW, Wissinger B, Kohl S, et al. CNGB3 achromatopsia with progressive loss of residual cone function and impaired rod-mediated function. Invest Ophthalmol Vis Sci 2007;48(8):3864–3871; doi: 10.1167/iovs.06-1521 17652762

[51] Kohl S, Varsanyi B, Antunes GA, et al. CNGB3 mutations account for 50% of all cases with autosomal recessive achromatopsia. Eur J Hum Genet 2005;13(3):302–308; doi: 10.1038/sj.ejhg.5201269 15657609

[52] Wissinger B, Gamer D, Jägle H, et al. CNGA3 mutations in hereditary cone photoreceptor disorders. Am J Hum Genet 2001;69(4):722–737; doi: 10.1086/323613 11536077 PMC1226059

[53] Fischer MD, Michalakis S, Wilhelm B, et al. Safety and vision outcomes of subretinal gene therapy targeting cone photoreceptors in achromatopsia: A nonrandomized controlled trial. JAMA Ophthalmol 2020;138(6):643–651; doi: 10.1001/jamaophthalmol.2020.1032 32352493 PMC7193523

[54] Molz B, Herbik A, Baseler HA, et al. Structural changes to primary visual cortex in the congenital absence of cone input in achromatopsia. Neuroimage Clin 2022;33:102925; doi: 10.1016/j.nicl.2021.102925 PMC8718719 34959047

[55] Vervoort R, Lennon A, Bird AC, et al. Mutational hot spot within a new RPGR exon in X-linked retinitis pigmentosa. Nat Genet 2000;25(4):462–466; doi: 10.1038/78182 10932196

[56] Cehajic-Kapetanovic J, Xue K, Martinez-Fernandez de la Camara C, et al. Initial results from a first-in-human gene therapy trial on X-linked retinitis pigmentosa caused by mutations in RPGR. Nat Med 2020;26(3):354–359; doi: 10.1038/s41591-020-0763-1 32094925 PMC7104347

[57] Tsang SH, Burns ME, Calvert PD, et al. Role for the target enzyme in deactivation of photoreceptor G protein in vivo. Science 1998;282(5386):117–121; doi: 10.1126/science.282.5386.117 9756475

[58] Dryja TP, Rucinski DE, Chen SH, et al. Frequency of mutations in the gene encoding the alpha subunit of rod cGMP-phosphodiesterase in autosomal recessive retinitis pigmentosa. Invest Ophthalmol Vis Sci 1999;40(8):1859–1865. 10393062

[59] McLaughlin ME, Ehrhart TL, Berson EL, et al. Mutation spectrum of the gene encoding the beta subunit of rod phosphodiesterase among patients with autosomal recessive retinitis pigmentosa. Proc Natl Acad Sci U S A 1995;92(8):3249–3253; doi: 10.1073/pnas.92.8.3249 7724547 PMC42143

[60] Pichard V, Provost N, Mendes-Madeira A, et al. AAV-mediated gene therapy halts retinal degeneration in PDE6β-deficient dogs. Mol Ther 2016;24(5):867–876; doi: 10.1038/mt.2016.37 26857842 PMC4881772

[61] Petit L, Lhériteau E, Weber M, et al. Restoration of vision in the pde6β-deficient dog, a large animal model of rod-cone dystrophy. Mol Ther 2012;20(11):2019–2030; doi: 10.1038/mt.2012.134 22828504 PMC3498794

[62] Feng W, Yasumura D, Matthes MT, et al. Mertk triggers uptake of photoreceptor outer segments during phagocytosis by cultured retinal pigment epithelial cells. J Biol Chem 2002;277(19):17016–17022; doi: 10.1074/jbc.M107876200 11861639

[63] Ghazi NG, Abboud EB, Nowilaty SR, et al. Treatment of retinitis pigmentosa due to MERTK mutations by ocular subretinal injection of adeno-associated virus gene vector: Results of a phase I trial. Hum Genet 2016;135(3):327–343; doi: 10.1007/s00439-016-1637-y 26825853

[64] Maguire AM, Bennett J, Aleman EM, et al. Clinical perspective: Treating RPE65-associated retinal dystrophy. Mol Ther 2021;29(2):442–463; doi: 10.1016/j.ymthe.2020.11.029 33278565 PMC7854308

[65] Saari JC, Crabb JW. Focus on molecules: Cellular retinaldehyde-binding protein (CRALBP). Exp Eye Res 2005;81(3):245–246; doi: 10.1016/j.exer.2005.06.015 16085009

[66] Fleisch VC, Schonthaler HB, von Lintig J, et al. Subfunctionalization of a retinoid-binding protein provides evidence for two parallel visual cycles in the cone-dominant zebrafish retina. J Neurosci 2008;28(33):8208–8216; doi: 10.1523/JNEUROSCI.2367-08.2008 18701683 PMC6670567

[67] Mitsios A, Dubis AM, Moosajee M. Choroideremia: From genetic and clinical phenotyping to gene therapy and future treatments. Ther Adv Ophthalmol 2018;10:2515841418817490; doi: 10.1177/2515841418817490 PMC6311551 30627697

[68] Furgoch MJ, Mewes-Arès J, Radziwon A, et al. Molecular genetic diagnostic techniques in choroideremia. Mol Vis 2014;20:535–544. 24791138 PMC4000712

[69] Preising M, Ayuso C. Rab escort protein 1 (REP1) in intracellular traffic: A functional and pathophysiological overview. Ophthalmic Genet 2004;25(2):101–110; doi: 10.1080/13816810490514333 15370541

[70] van den Hurk JA, Schwartz M, van Bokhoven H, et al. Molecular basis of choroideremia (CHM): Mutations involving the Rab escort protein-1 (REP-1) gene. Hum Mutat 1997;9(2):110–117; doi: 10.1002/(SICI)1098-1004(1997)9:2<110::AID-HUMU2>3.0.CO;2-D 9067750

[71] Flannery JG, Bird AC, Farber DB, et al. A histopathologic study of a choroideremia carrier. Invest Ophthalmol Vis Sci 1990;31(2):229–236; 10.1002/(SICI)1098-1004(1997)9:2<110::AID-HUMU2>3.0.CO;2-D 2303326

[72] Renner AB, Fiebig BS, Cropp E, et al. Progression of retinal pigment epithelial alterations during long-term follow-up in female carriers of choroideremia and report of a novel CHM mutation. Arch Ophthalmol 2009;127(7):907–912; doi: 10.1001/archophthalmol.2009.123 19597113

[73] Tolmachova T, Anders R, Abrink M, et al. Independent degeneration of photoreceptors and retinal pigment epithelium in conditional knockout mouse models of choroideremia. J Clin Invest 2006;116(2):386–394; doi: 10.1172/JCI26617 16410831 PMC1326146

[74] Lam BL, Davis JL, Gregori NZ. Choroideremia gene therapy. Int Ophthalmol Clin 2021;61(4):185–193; doi: 10.1097/IIO.0000000000000385 34584056 PMC8478312

[75] Patrício MI, Barnard AR, Orlans HO, et al. Inclusion of the Woodchuck hepatitis virus posttranscriptional regulatory element enhances AAV2-driven transduction of mouse and human retina. Mol Ther Nucleic Acids 2017;6:198–208; doi: 10.1016/j.omtn.2016.12.006 28325286 PMC5363497

[76] MacLaren RE, Groppe M, Barnard AR, et al. Retinal gene therapy in patients with choroideremia: Initial findings from a phase ½ clinical trial. Lancet 2014;383(9923):1129–1137; doi: 10.1016/S0140-S6736(13)62117-0 24439297 PMC4171740

[77] Edwards TL, Jolly JK, Groppe M, et al. Visual acuity after retinal gene therapy for choroideremia. N Engl J Med 2016;374(20):1996–1998; doi: 10.1056/NEJMc1509501 27120491 PMC4996318

[78] Dimopoulos IS, Hoang SC, Radziwon A, et al. Two-year results after AAV2-mediated gene therapy for choroideremia: The Alberta experience. Am J Ophthalmol 2018;193:130–142; doi: 10.1016/j.ajo.2018.06.011 29940166

[79] Lam BL, Davis JL, Gregori NZ, et al. Choroideremia gene therapy phase 2 clinical trial: 24-month results. Am J Ophthalmol 2019;197:65–73; doi: 10.1016/j.ajo.2018.09.012 30240725

[80] Fischer MD, Ochakovski GA, Beier B, et al. Efficacy and safety of retinal gene therapy using adeno-associated virus vector for patients with choroideremia: A randomized clinical trial. JAMA Ophthalmol 2019;137(11):1247–1254; doi: 10.1001/jamaophthalmol.2019.3278 31465092 PMC6865291

[81] Aleman TS, Huckfeldt RM, Serrano LW, et al. AAV2-hCHM subretinal delivery to the macula in choroideremia: Two year interim results of an ongoing phase I/II gene therapy trial. Ophthalmology 2022;S0161-6420(22)00438-9. [Epub ahead of print]; doi: 10.1016/j.ophtha.2022.06.006 35714735

[82] Kumaran N, Moore AT, Weleber RG, et al. Leber congenital amaurosis/early-onset severe retinal dystrophy: Clinical features, molecular genetics and therapeutic interventions. Br J Ophthalmol 2017;101(9):1147–1154; doi: 10.1136/bjophthalmol-2016-309975 28689169 PMC5574398

[83] den Hollander AI, Roepman R, Koenekoop RK, et al. Leber congenital amaurosis: Genes, proteins and disease mechanisms. Prog Retin Eye Res 2008;27(4):391–419; doi: 10.1016/j.preteyeres.2008.05.003 18632300

[84] Gu SM, Thompson DA, Srikumari CR, et al. Mutations in RPE65 cause autosomal recessive childhood-onset severe retinal dystrophy. Nat Genet 1997;17(2):194–197; doi: 10.1038/ng1097-ng194 9326941

[85] den Hollander AI, Koenekoop RK, Yzer S, et al. Mutations in the CEP290 (NPHP6) gene are a frequent cause of Leber congenital amaurosis. Am J Hum Genet 2006;79(3):556–561; doi: 10.1086/507318 16909394 PMC1559533

[86] Khanna H. Photoreceptor sensory cilium: Traversing the ciliary gate. Cells 2015;4(4):674–686; doi: 10.3390/cells4040674 26501325 PMC4695852

[87] Zhang W, Li L, Su Q, et al. Gene therapy using a miniCEP290 fragment delays photoreceptor degeneration in a mouse model of Leber congenital amaurosis. Hum Gene Ther 2018;29(1):42–50; doi: 10.1089/hum.2017.049 28679290 PMC5770090

[88] Adli M. The CRISPR tool kit for genome editing and beyond. Nat Commun 2018;9(1):1911; doi: 10.1038/s41467-018-04252-2 PMC5953931 29765029

[89] Tantri A, Vrabec TR, Cu-Unjieng A, et al. X-linked retinoschisis: A clinical and molecular genetic review. Surv Ophthalmol 2004;49(2):214–230; doi: 10.1016/j.survophthal.2003.12.007 14998693

[90] Sauer CG, Gehrig A, Warneke-Wittstock R, et al. Positional cloning of the gene associated with X-linked juvenile retinoschisis. Nat Genet 1997;17(2):164–170; doi: 10.1038/ng1097-ng164 9326935

[91] Rao P, Dedania VS, Drenser KA. Congenital X-linked retinoschisis: An updated clinical review. Asia Pac J Ophthalmol (Phila) 2018;7(3):169–175; doi: 10.22608/APO.201803 29633586

[92] Falcone PM, Brockhurst RJ. X-chromosome-linked juvenile retinoschisis: Clinical aspects and genetics. Int Ophthalmol Clin 1993;33(2):193–202; doi: 10.1097/00004397-199303320-00018 8325733

[93] Bush RA, Wei LL, Sieving PA. Convergence of human genetics and animal studies: Gene therapy for X-linked retinoschisis. Cold Spring Harb Perspect Med 2015;5(8):a017368; doi: 10.1101/cshperspect.a017368 PMC4526726 26101206

[94] Molday LL, Hicks D, Sauer CG, et al. Expression of X-linked retinoschisis protein RS1 in photoreceptor and bipolar cells. Invest Ophthalmol Vis Sci 2001;42(3):816–825. 11222545

[95] Cukras C, Wiley HE, Jeffrey BG, et al. Retinal AAV8-RS1 gene therapy for X-linked retinoschisis: Initial findings from a phase I/IIa trial by intravitreal delivery. Mol Ther 2018;26(9):2282–2294; doi: 10.1016/j.ymthe.2018.05.025 30196853 PMC6127971

[96] Mackey DA, Oostra RJ, Rosenberg T, et al. Primary pathogenic mtDNA mutations in multigeneration pedigrees with Leber hereditary optic neuropathy. Am J Hum Genet 1996;59(2):481–485. 8755941 PMC1914749

[97] Yu-Wai-Man P, Turnbull DM, Chinnery PF. Leber hereditary optic neuropathy. J Med Genet 2002;39(3):162–169; doi: 10.1136/jmg.39.3.162 11897814 PMC1735056

[98] Carelli V, La Morgia C, Valentino ML, et al. Retinal ganglion cell neurodegeneration in mitochondrial inherited disorders. Biochim Biophys Acta 2009;1787(5):518–528; doi: 10.1016/j.bbabio.2009.02.024 19268652

[99] Bahr T, Welburn K, Donnelly J, et al. Emerging model systems and treatment approaches for Leber's hereditary optic neuropathy: Challenges and opportunities. Biochim Biophys Acta Mol Basis Dis 2020;1866(6):165743; doi: 10.1016/j.bbadis.2020.165743 PMC9252426 32105823

[100] Nikoskelainen EK, Huoponen K, Juvonen V, et al. Ophthalmologic findings in Leber hereditary optic neuropathy, with special reference to mtDNA mutations. Ophthalmology 1996;103(3):504–514; doi: 10.1016/s0161-s6420(96)30665-9 8600429

[101] Yen M-Y, Wang A-G, Wei Y-H. Leber's hereditary optic neuropathy: A multifactorial disease. Prog Retin Eye Res 2006;25(4):381–396; doi: 10.1016/j.preteyeres.2006.05.002 16829155

[102] Guy J, Qi X, Pallotti F, et al. Rescue of a mitochondrial deficiency causing Leber Hereditary Optic Neuropathy. Ann Neurol 2002;52(5):534–542; doi: 10.1002/ana.10354 12402249

[103] Carelli V, Ross-Cisneros FN, Sadun AA. Mitochondrial dysfunction as a cause of optic neuropathies. Prog Retin Eye Res 2004;23(1):53–89; doi: 10.1016/j.preteyeres.2003.10.003 14766317

[104] Wallace DC, Singh G, Lott MT, et al. Mitochondrial DNA mutation associated with Leber's Hereditary Optic Neuropathy. Science 1988;242(4884):1427–1430; doi: 10.1126/science.3201231 3201231

[105] Guy J, Feuer WJ, Davis JL, et al. Gene therapy for Leber Hereditary Optic Neuropathy: Low- and medium-dose visual results. Ophthalmology 2017;124(11):1621–1634; doi: 10.1016/j.ophtha.2017.05.016 28647203 PMC5831379

[106] Feuer WJ, Schiffman JC, Davis JL, et al. Gene therapy for Leber Hereditary Optic Neuropathy: Initial results. Ophthalmology 2016;123(3):558–570; doi: 10.1016/j.ophtha.2015.10.025 26606867 PMC5287094

[107] Bouquet C, Vignal Clermont C, Galy A, et al. Immune response and intraocular inflammation in patients with leber hereditary optic neuropathy treated with intravitreal injection of recombinant adeno-associated virus 2 carrying the ND4 gene: A secondary analysis of a phase ½ clinical trial. JAMA Ophthalmol 2019;137(4):399–406; doi: 10.1001/jamaophthalmol.2018.6902 30730541 PMC6459107

[108] Vignal C, Uretsky S, Fitoussi S, et al. Safety of rAAV2/2-ND4 gene therapy for Leber hereditary optic neuropathy. Ophthalmology 2018;125(6):945–947; doi: 10.1016/j.ophtha.2017.12.036 29426586

[109] Biousse V, Newman NJ, Yu-Wai-Man P, et al. Long-term follow-up after unilateral intravitreal gene therapy for leber hereditary optic neuropathy: The RESTORE Study. J Neuroophthalmol 2021;41(3):309–315; doi: 10.1097/WNO.0000000000001367 34415265 PMC8366761

[110] Newman NJ, Yu-Wai-Man P, Carelli V, et al. Efficacy and safety of intravitreal gene therapy for leber hereditary optic neuropathy treated within 6 months of disease onset. Ophthalmology 2021;128(5):649–660; doi: 10.1016/j.ophtha.2020.12.012 33451738

[111] Yu-Wai-Man P, Newman NJ, Carelli V, et al. Bilateral visual improvement with unilateral gene therapy injection for Leber hereditary optic neuropathy. Sci Transl Med 2020;12(573):eaaz7423; doi: 10.1126/scitranslmed.aaz7423 33298565

[112] Wan X, Pei H, Zhao M-J, et al. Efficacy and safety of rAAV2-ND4 treatment for Leber's's hereditary optic neuropathy. Sci Rep 2016;6:21587; doi: 10.1038/srep21587 PMC4759604 26892229

[113] Yang S, Ma S-Q, Wan X, et al. Long-term outcomes of gene therapy for the treatment of Leber's hereditary optic neuropathy. EBioMedicine 2016;10:258–268; doi: 10.1016/j.ebiom.2016.07.002 27426279 PMC5006665

[114] Pan Z-H, Lu Q, Bi A, et al. Optogenetic approaches to restoring vision. Annu Rev Vis Sci 2015;1:185–210; doi: 10.1146/annurev-vision-082114-035532 28532375

[115] Simunovic MP, Shen W, Lin JY, et al. Optogenetic approaches to vision restoration. Exp Eye Res 2019;178:15–26; doi: 10.1016/j.exer.2018.09.003 30218651

[116] Wright W, Gajjeraman S, Batabyal S, et al. Restoring vision in mice with retinal degeneration using multicharacteristic opsin. Neurophotonics 2017;4(4):041412; doi: 10.1117/1.NPh.4.4.041412 28840163

[117] Gauvain G, Akolkar H, Chaffiol A, et al. Optogenetic therapy: High spatiotemporal resolution and pattern discrimination compatible with vision restoration in non-human primates. Commun Biol 2021;4(1):125; doi: 10.1038/s42003-020-01594-w PMC7840970 33504896

[118] Sahel J-A, Boulanger-Scemama E, Pagot C, et al. Partial recovery of visual function in a blind patient after optogenetic therapy. Nat Med 2021;27(7):1223–1229; doi: 10.1038/s41591-021-01351-4 34031601

[119] Aiello LP; DCCT/EDIC Research Group. Diabetic retinopathy and other ocular findings in the diabetes control and complications trial/epidemiology of diabetes interventions and Complications Study. 2014;37(1):17–23; doi: 10.2337/dc13-dc2251 PMC3867989 24356593

[120] Miller K, Fortun JA. Diabetic macular edema: Current understanding, pharmacologic treatment options, and developing therapies. Asia Pac J Ophthalmol (Phila) 2018;7(1):28–35; doi: 10.22608/APO.2017529 29473719

[121] Constable IJ, Pierce CM, Lai C-M, et al. Phase 2a randomized clinical trial: safety and post hoc analysis of subretinal rAAV.sFLT-1 for wet age-related macular degeneration. EBioMedicine 2016;14(92–100):168–175; doi: 10.1016/j.ebiom.2016.11.016 27865764 PMC5161436

[122] Constable IJ, Lai CM, Magno AL, et al. Gene therapy in neovascular age-related macular degeneration: Three-year follow-up of a phase 1 randomized dose escalation trial. Am J Ophthalmol 2017;177(150–158); doi: 10.1016/j.ajo.2017.02.018 28245970

[123] Rakoczy EP, Lai CM, Magno AL, et al. Gene therapy with recombinant adeno-associated vectors for neovascular age-related macular degeneration: 1 year follow-up of a phase 1 randomised clinical trial. Lancet 2015;386(10011):2395–2403; doi: 10.1016/S0140-6736(15)00345-1 26431823

[124] Rakoczy EP, Magno AL, Lai CM, et al. Three-year follow-up of phase 1 and 2a rAAV.sFLT-1 subretinal gene therapy trials for exudative age-related macular degeneration. Am J Ophthalmol 2019;204(113–123); doi: 10.1016/j.ajo.2019.03.006 30878487

[125] Heier JS, Kherani S, Desai S, et al. Intravitreous injection of AAV2-sFLT01 in patients with advanced neovascular age-related macular degeneration: A phase 1, open-label trial. Lancet 2017;390(10089):50–61; doi: 10.1016/s0140-s6736(17)30979-0 28526489

[126] Friedman DS, O'Colmain BJ, Munoz B, et al. Prevalence of age-related macular degeneration in the United States. Arch Ophthalmol 2004;122(4):564–572; doi: 10.1001/archopht.122.4.564 15078675

[127] Geerlings MJ, de Jong EK, den Hollander AI. The complement system in age-related macular degeneration: A review of rare genetic variants and implications for personalized treatment. Mol Immunol 2017;84:65–76; doi: 10.1016/j.molimm.2016.11.016 27939104 PMC5380947

[128] Reynolds R, Rosner B, Seddon JM. Dietary omega-3 fatty acids, other fat intake, genetic susceptibility, and progression to incident geographic atrophy. Ophthalmology 2013;120(5):1020–1028; doi: 10.1016/j.ophtha.2012.10.020 23481534 PMC3758110

[129] Seddon JM, George S, Rosner B. Cigarette smoking, fish consumption, omega-3 fatty acid intake, and associations with age-related macular degeneration: The US Twin Study of Age-Related Macular Degeneration. Arch Ophthalmol 2006;124(7):995–1001; doi: 10.1001/archopht.124.7.995 16832023

[130] Fritsche LG, Igl W, Bailey JN, et al. A large genome-wide association study of age-related macular degeneration highlights contributions of rare and common variants. Nat Genet 2016;48(2):134–143; doi: 10.1038/ng.3448 26691988 PMC4745342

[131] Cheng S-Y, Cipi J, Ma S, et al. Altered photoreceptor metabolism in mouse causes late stage age-related macular degeneration-like pathologies. Proc Natl Acad Sci U S A 2020;117(23):13094–13104; doi: 10.13039/100006312 32434914 PMC7293639

[132] Cabral de Guimaraes TA, Daich Varela M, Georgiou M, et al. Treatments for dry age-related macular degeneration: Therapeutic avenues, clinical trials and future directions. Br J Ophthalmol 2022;106(3):297–304; doi: 10.13039/501100017645 33741584 PMC8867261

[133] Kawa MP, Machalinska A, Roginska D, et al. Complement system in pathogenesis of AMD: Dual player in degeneration and protection of retinal tissue. J Immunol Res 2014;2014(7):1–12; doi: 10.1155/2014/483960 PMC4168147 25276841

[134] Anderson DH, Mullins RF, Hageman GS, et al. A role for local inflammation in the formation of drusen in the aging eye. Am J Ophthalmol 2002;134(3):411–431; doi: 10.1016/s0002-s9394(02)01624-0 12208254

[135] Sugita Y, Masuho Y. CD59: Its role in complement regulation and potential for therapeutic use. Immunotechnology 1995;1(3–4):157–168; doi: 10.1016/1380-2933(95)00018-6 9373344

[136] Dugel PU. CLINICAL TRIAL DOWNLOAD: Data on a gene therapy for dry and wet AMD. Retin Physician 2020;17(April 2020):16–17.

[137] Dreismann AK, McClements ME, Barnard AR, et al. Functional expression of complement factor I following AAV-mediated gene delivery in the retina of mice and human cells. Gene Ther 2021;28(5):265–276; doi: 10.13039/501100013373 33750925 PMC8149295

[138] Gupta A, Kafetzis KN, Tagalakis AD, et al. RNA therapeutics in ophthalmology—Translation to clinical trials. Exp Eye Res 2021;205(1):108482; doi: 10.13039/501100000265 33548256

[139] Ghoraba HH, Akhavanrezayat A, Karaca I, et al. Ocular gene therapy: A literature review with special focus on immune and inflammatory responses. Clin Ophthalmol 2022;16(1753–1771); doi: 10.2147/OPTH.S364200 PMC9173725 35685379

[140] Cheng S-Y, Punzo C. Ocular inflammation with anti-vascular endothelial growth factor treatments. Hum Gene Ther 2021;32(13–14):639–641; doi: 10.1089/hum.2021.29167.syc 34283642

